# Circulatory amino acid responses to milk consumption in dairy and lactose intolerant individuals

**DOI:** 10.1038/s41430-022-01119-0

**Published:** 2022-04-22

**Authors:** Utpal Kumar Prodhan, Amber Marie Milan, Aahana Shrestha, Mark Hedley Vickers, David Cameron-Smith, Matthew Philip Greig Barnett

**Affiliations:** 1 Liggins Institute, The University of Auckland, 85 Park Road, Grafton, Private Bag 92019, Auckland, 1023 New Zealand; 2 The Riddet Institute, Palmerston North, 4442 New Zealand; 3 Department of Food Technology and Nutritional Science, Mawlana Bhashani Science and Technology University, Tangail, 1902 Bangladesh; 4 Smart Foods Innovation Centre of Excellence, AgResearch Limited, Private Bag 11008, Palmerston North, 4442 New Zealand; 5 The High-Value Nutrition National Science Challenge, Auckland, 1023 New Zealand; 6 Singapore Institute for Clinical Sciences, Agency for Science, Technology, and Research, Singapore, 117609 Singapore

**Keywords:** Metabolism, Digestive signs and symptoms, Nutrition

## Abstract

**Background/objectives:**

Self-reported digestive intolerance to dairy foods is common. As dairy can be an important source of dietary protein, this study aimed to identify whether milk protein digestion is compromised in individuals with digestive intolerance.

**Subjects/methods:**

Adult women (*n* = 40) were enroled in this double-blinded, randomised cross-over trial, with digestive symptoms characterised using a lactose challenge and self-reported digestive symptom questionnaire. Participants were classified as either lactose intolerant (LI, *n* = 10), non-lactose dairy intolerant (NLDI, *n* = 20) or dairy tolerant (DT, *n* = 10). In a randomised sequence, participants consumed three different kinds of milk (750 ml); conventional milk (CON), a2 Milk™ (A2M), and lactose-free conventional milk (LF-CON). Circulatory plasma amino acid (AA) concentrations were measured at baseline and every 30 min until 3 h post-ingestion.

**Results:**

In all participants across all milk types, plasma AA concentrations (AUC_0-180_) increased after milk ingestion with no significant differences in responses observed between milk types or participants (*P* > 0.05), with the exception of the suppressed lysine response in the DT group following A2M ingestion, relative to the other two groups and milk types (*P* < 0.05).

**Conclusion:**

Milk protein digestion, as determined by circulatory AAs, is largely unaffected by dairy- and lactose- intolerances.

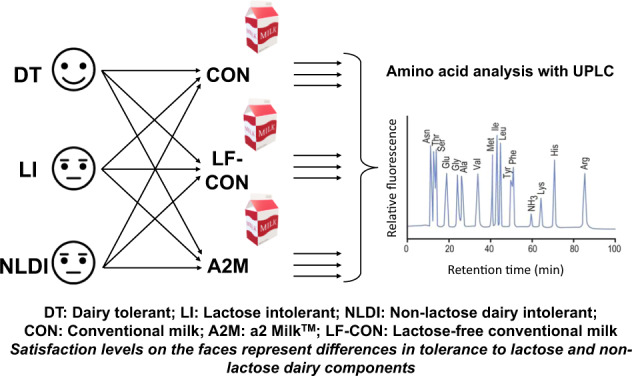

## Introduction

Lactose intolerance is a common complaint, affecting about 65–70% of adults globally [1]. For the majority of adults, the intolerance symptoms can be ascribed to single-nucleotide polymorphisms (SNPs) in the gene region of the small intestinal β-lactase enzyme [2, 3]. For individuals with SNP variants that suppress β-lactase expression after infancy, lactose is minimally digested in the small intestine. Instead, lactose enters the large intestine where its rapid fermentation may result in the acute adverse gastrointestinal (GI) events that are symptomatically typical of lactose intolerance [2, 4]. However, this is not the only form of dairy intolerance that may be experienced. There is a proportion of the adult population where lactose malabsorption does not appear to be the sole cause [5, 6], but the causative factor has not yet been identified.

It has been postulated that individual dairy proteins may also elicit symptoms of intolerance [7]. Particular attention has focused on the β-casein proteins, which for most commercial bovine milk types contain two major isoforms (A1 and A2). These differ by a single amino acid at position 67 in their protein structure. Hypothetically, the A1 isoform will generate a bioactive peptide, beta-casomorphin-7 (BCM-7) [8–10], whilst the A2 isoform will not. BCM-7 has been proposed as a regulator of GI function, including delaying gastric transit, promoting intestinal inflammation and aggravating GI symptoms in experimental models [8] and in human cohorts [11]. Therefore, it is possible that in some individuals, the presence of A1 β-casein and the liberation of BCM-7 may impact on GI function to elicit symptoms of milk intolerance [9].

Whilst it is known that impaired protein digestion is evident in individuals with intestinal diseases, including inflammatory bowel diseases such as Crohn’s disease, and coeliac disease [12–14], less is known of the disturbances in milk protein digestion in lactose and dairy intolerance where altered motility, visceral hypersensitivity and inflammation may also be evident [15]. Therefore, we hypothesised that in lactose intolerant (LI) individuals, bovine milk types containing lactose, irrespective of the β-casein isoform, would negatively impact on protein digestion. In contrast, in those individuals identified as exhibiting non-lactose dairy intolerance (NLDI), the presence of lactose would not alter protein digestion. We also hypothesised that for the bovine milk types containing A1 β-casein, protein digestion would be delayed in NLDI individuals. Finally, for dairy tolerant (DT) individuals, we hypothesised that they would comfortably digest protein following ingestion of all the bovine milk types; however, the compositional and microstructural variations between the milk types would result in a difference in protein digestion rate.

## Methods

### Subjects

This paper reports on secondary outcomes from a clinical study that was conducted with the primary aim of investigating the impact of bovine milk beta-casein variants on digestive comfort. The methodology utilised in this clinical study has been described elsewhere [16]. In brief, exclusion criteria were set to ensure participants had an absence of current or past history of GI diseases and were free from cardio-metabolic diseases. Following preliminary screening, healthy young women (*n* = 59) aged 20–30 years were recruited. The study was conducted according to the guidelines laid out by the Declaration of Helsinki. All procedures involving human subjects were approved by the Southern Health and Disability Ethics Committee (New Zealand, 16/STH/175), and all participants provided written informed consent. This study was registered with the Australian New Zealand Clinical Trials Registry at www.anzctr.org.au (ACTRN12616001694404).

### Study design and intervention

This study utilised a double-blind randomised cross-over design. Participants attended the Nutrition and Mobility Clinic at the University of Auckland on four occasions between January and May 2017, separated by at least 1 week. After primary screening with a validated symptom questionnaire [17] to screen for lactose intolerance, a lactose challenge (50 g lactose in 250 ml water) [18] was conducted. Following lactose ingestion, subjects were stratified into three groups (LI, NLDI and DT) based on their perceived symptom score, hydrogen breath test and homoeostatic plasma glucose level as previously described [16]. Progressive recruitment continued until 10 LI, 20 NLDI and 10 DT subjects were identified. As NLDI was defined on the absence of lactose intolerance rather than pre-defined clinical criteria, we recruited NLDI subjects in a ratio of 2:1 corresponding to DT and LI subjects to account for possible divergent NLDI phenotypes.

**Fig. 1:**
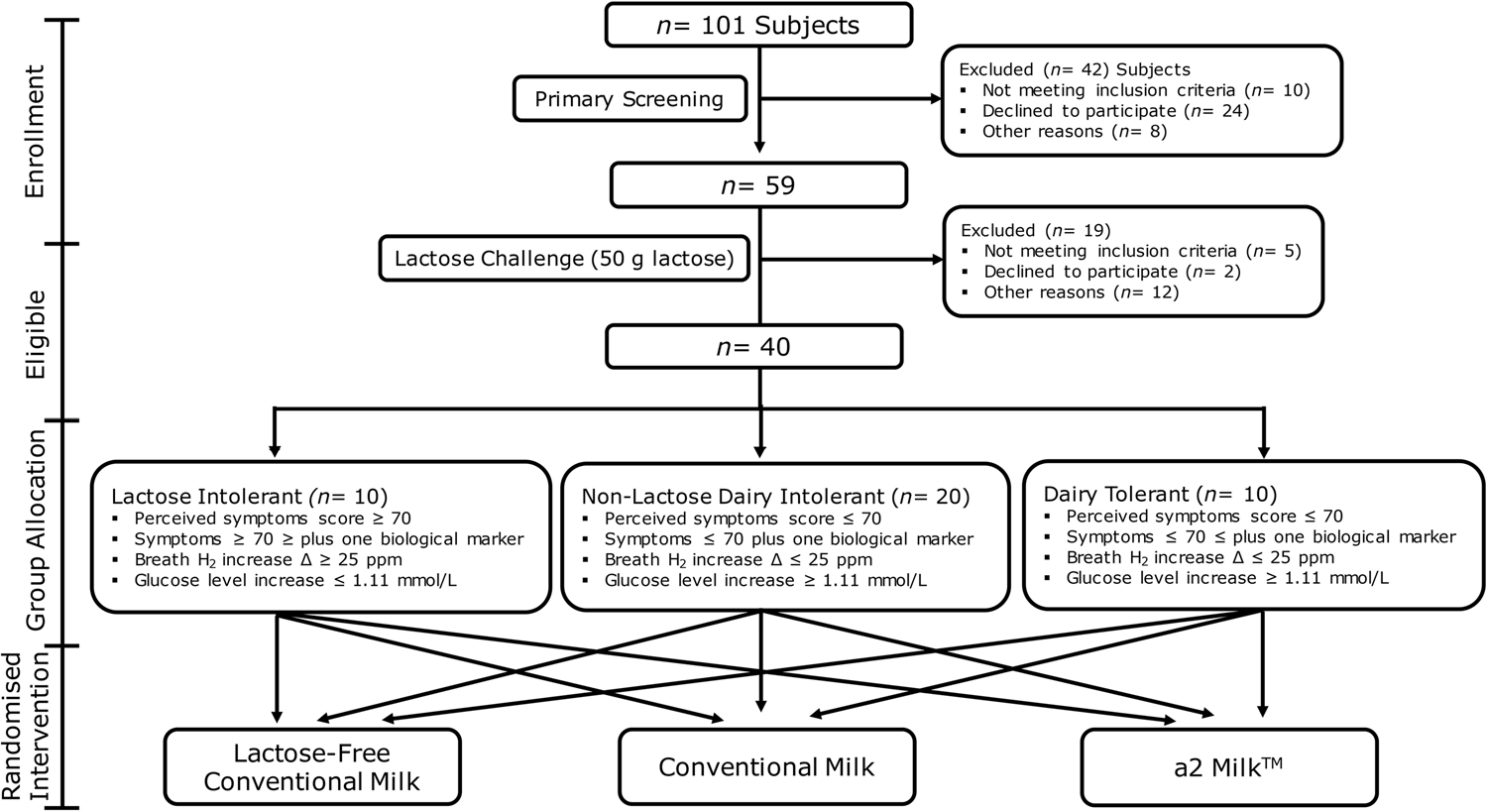
Flow diagram of the study design. The figure shows participant recruitment, grouping criteria, and randomised intervention.

Eligibile participants were then allocated to a randomised crossover sequence (www.randomizer.org) concealed in sealed envelopes to drink the following three ultra-high temperature (UHT) treated bovine milk types: lactose-free conventional milk (LF-CON; containing A1 and A2 β-casein, with enzymatically hydrolysed lactose; Woolworths, Sydney, Australia), conventional milk (CON; containing lactose, A1 and A2 β-casein; UHT Blue Top Longlife Milk, Anchor™, Auckland, New Zealand) and a2 Milk™ (A2M; containing lactose and only A2 β-casein but no A1 β-casein; a2 Milk™ Full Cream Milk, The a2 Milk™ Company Limited, Sydney, Australia). Dietary instructions, restrictions and supply provided to the participants have been described previously [16]. Participants were asked to drink 750 ml of milk in a single session to go beyond the lactose tolerable capacity of LI subjects [9]. Participant enrolment and the randomised intervention, including the group classification criteria, are presented in Fig. 1.

### Collection and preparation of blood samples

During each clinic visit for lactose or milk challenges, fasting venous blood samples were collected. Postprandial blood samples were collected over a period of 3 h at 30 min intervals after drinking the assigned milk. Blood samples were collected in vacutainers (Becton Dickinson & Company, Auckland, New Zealand) for serum and plasma (EDTA). Serum tubes were allowed to clot for 15 min at room temperature, while plasma tubes were kept at 4 °C, before centrifugation (2000 × *g* for 15 min, 4 °C). Supernatants were collected and stored at −20 °C prior to analyses.

### Measurement of biochemical variables and plasma free amino acid concentrations

Standard enzymatic colorimetric assays were used to analyse plasma glucose and triglycerides using a Cobas c311 clinical chemistry analyser (Roche Diagnostics, Basal, Switzerland). Serum insulin was measured by electrochemiluminescence immunoassay using a Cobas e411 immunoassay analyser (Roche Diagnostics, Basal, Switzerland).

Plasma free amino acids (AA) were assessed using ultra-high pressure liquid chromatography following standard protocols [19, 20]. Briefly, plasma samples were extracted with the addition of sulphuric acid followed by tungstate precipitation. Pre column fluorescent derivatisation was achieved by adding borate buffer and tagging with 6-aminoquinolyl N hydroxysuccinimidyl carbamate (AccQ) reagent. The mixture was then injected through a Kinetex EVO C18 1.7 µm 150 × 2.1 mm separation column (Dionex Ultimate 3000 pump; Thermo Fisher Scientific, Dornierstrasse, Germany) coupled with a fluorescence detector. L-norvaline was used as an internal standard. Chromeleon 7.1 software (Thermo Fisher Scientific) was used to extract data directly from the spectrum based on the standard curves produced from mixed standards (Sigma Chemical Company, St. Louis, MO, USA).

### Milk composition analysis

The A1 β-casein protein was separated using a high pressure liquid chromatography (HPLC) system with a Hypersil Gold C18 (2.1 × 100 mm, 1.9 μm) column (Thermo Scientific, Dornierstrasse, Germany), coupled to a TSQ Quantiva triple-quadrupole mass spectrometer (Thermo Scientific, USA) using a selected reaction monitoring (SRM) method where the samples were enzyme digested with internal standards labelled specifically for A1 β-casein (Biomatik, Ontario, Canada). Measurement of A1 β-casein peptide was conducted in triplicate on each sample.

AA composition of the milk samples was determined by RP-HPLC with fluorescence detector following AOAC 994.12 method with some modification [21]. The samples were HCl acid extracted followed by pre-column derivatisation with AccQ Tag. Cysteine and methionine were determined with performic acid oxidation, whereas tryptophan content was analysed with alkaline hydrolysis. Detailed milk composition is presented in Table 1.

**Table 1:** Nutritional composition per milk serving (750 ml).

Nutrient	Unit	LF-CON	A2M	CON
Energy	kJ	1935	2063	2010
Protein	g	25.5	24.8	26.3
Total fat	g	25.5	26.3	25.5
Saturated fat	g	18.0	18.0	17.3
Total carbohydrate	g	33.0	37.5	36.0
Lactose	g	Not detected	35.3	36.0
Calcium	mg	900	817	915
A1 β-casein^a^	% total β-casein	23.5	0	21.6
Amino acids^b^				
Valine	g	1.68	1.53	1.38
Leucine	g	2.48	2.41	2.11
Isoleucine	g	1.31	1.24	1.09
Phenylalanine	g	1.24	1.17	1.09
Methionine	g	0.66	0.66	0.58
Lysine	g	1.90	1.90	1.75
Histidine	g	0.66	0.58	0.58
Threonine	g	1.17	1.02	0.95
Tryptophan	g	0.22	0.29	0.29
Glycine	g	0.58	0.51	0.51
Alanine	g	0.80	0.80	0.73
Arginine	g	0.87	0.87	0.80
Serine	g	1.31	1.24	1.09
Proline	g	2.55	2.48	2.19
Tyrosine	g	1.31	1.24	1.09
Aspartic acid	g	2.04	1.90	1.68
Glutamic acid	g	5.39	5.03	4.37
Cysteine	g	0.22	0.15	0.15

Unless otherwise stated, values are as provided on the nutrition information panel (NIP).

*LF-CON* lactose-free conventional milk, *CON* conventional milk containing both A1 and A2 β-casein, *A2M* milk containing exclusively A2 β-casein.

^a^A1 β-casein values are measured by LC-MS. Mean of three replicates.

^b^Amino acids are measured by RP-HPLC.

### Statistical analysis

Based on the effect size and variance observed in a previous study in our laboratory examining the rate of protein digestion and absorption by measuring plasma branched-chain amino acid (BCAA) concentrations (mean ± SD) at 30 min post-ingestion [22], it was calculated that 10 subjects per group would provide more than 80% power to detect differences with an alpha level of 5%. Data were analysed with linear mixed-effects AR(1): Heterogeneous model (time, groups and milk-types) followed by Sidak adjusted multiple comparison post hoc tests using SPSS Statistics 25 (IBM Corp., Armonk, New York, USA). The baseline-adjusted incremental area under the curve (iAUC_0-180_) was calculated and compared between the groups using two-factor ANOVA. Baseline subject characteristics were compared between the groups using a Univariate General Linear Model. Alpha was set at 0.05. Three times the interquartile range were used to detect and eliminate statistical outliers and linear mixed-effects model analysis was used to account for missing data points. GraphPad Prism version 8 for Windows (GraphPad Software, San Diego, CA, USA) was used for generating figures. Unless otherwise stated, data are presented as mean ± SEM.

## Results

Baseline subject characteristics including age, ethnicity and body mass index of the participants have been reported previously [16]. Other baseline clinical characteristics of participants by study groups are provided in Table 2, with the participants characterised into the three study groups (LI, NLDI and DT).

**Table 2:** Baseline subject characteristics.

	LI	NLDI	DT	*P* value
*N*	10	20	10	
Age (Years)	27 ± 1	26 ± 1	25 ± 1	0.47
BMI	22.9 ± 0.9	22.5 ± 0.5	24.5 ± 1.1	0.15
TAG (mmol/l)	1.17 ± 0.16	1.08 ± 0.09	1.16 ± 0.15	0.85
Glucose (mmol/l)	5.48 ± 0.35	5.37 ± 0.19	5.47 ± 0.14	0.92
Insulin (mU/l)	10.89 ± 1.26	8.07 ± 0.83	12.08 ± 2.10	0.07
HOMA-IR	2.54 ± 0.35	1.99 ± 0.25	2.91 ± 0.47	0.14

Values represent mean ± SEM. TAG and glucose were measured in plasma, whereas insulin was measured in serum.

*BMI* body mass index, *TG* triglyceride, *HOMA-IR* homoeostatic model assessment for insulin resistance.

The baseline AA plasma profile of participants by study groups is provided in Table 3. Compared to the NLDI group, the LI group had a higher baseline concentration of tyrosine (*P* = 0.022) and glutamic acid (*P* = 0.018); while compared to the DT group, the LI group had higher baseline concentration of tyrosine (*P* = 0.021), glutamic acid (*P* = 0.047), arginine (*P* = 0.042), taurine (*P* = 0.041) and hydroxyproline (*P* = 0.017). Also, in contrast to the DT group, the NLDI group had a higher baseline concentration of histidine (*P* = 0.002) and citrulline (*P* = 0.019). All *P* values for comparisons for which there was a significant difference are highlighted with bold text.

**Table 3:** Baseline plasma amino acid concentrations in study participants.

	LI (*n* = 10)	NLDI (*n* = 20)	DT (*n* = 10)	*P value*
*Branched-chain amino acids*				
Valine	211.45 ± 7.40	204.00 ± 4.53	198.40 ± 7.12	0.400
Leucine	101.82 ± 2.69	97.66 ± 2.09	94.39 ± 3.23	0.206
Isoleucine	59.80 ± 2.01	55.91 ± 1.28	54.23 ± 2.20	0.119
*Other essential amino acids*				
Phenylalanine	103.08 ± 3.14	98.87 ± 3.44	89.01 ± 3.76	0.052
Methionine	23.93 ± 0.92	25.70 ± 1.03	23.06 ± 1.06	0.184
Lysine	67.97 ± 2.43	63.30 ± 1.29	65.22 ± 2.74	0.240
Histidine	44.99 ± 1.17	47.63 ± 1.47^a^	40.42 ± 1.24	**0.003**
Threonine	129.74 ± 5.96	139.14 ± 5.98	131.62 ± 7.64	0.539
*Non-essential amino acids*				
Glycine	224.09 ± 16.74	207.81 ± 9.32	197.79 ± 10.10	0.375
Asparagine	35.43 ± 2.05	39.79 ± 1.30	35.78 ± 1.16	0.062
Alanine	287.66 ± 13.30	272.87 ± 8.56	263.11 ± 9.75	0.337
Arginine	71.10 ± 5.97^c^	65.13 ± 3.47	53.73 ± 3.79	**0.042**
Serine	109.36 ± 4.75	108.77 ± 3.10	107.61 ± 3.31	0.956
Proline	195.26 ± 24.06	231.78 ± 20.02	201.78 ± 21.29	0.424
Tyrosine	58.15 ± 3.28^bc^	49.32 ± 1.72	47.93 ± 2.35	**0.011**
Aspartic acid	10.98 ± 0.61	10.31 ± 0.52	8.87 ± 0.61	0.075
Glutamic acid	119.04 ± 6.75^bc^	99.17 ± 3.91	99.03 ± 5.33	**0.014**
Glutamine	353.24 ± 15.83	361.58 ± 10.12	340 ± 12.82	0.472
*Non-proteogenic amino acids*				
Taurine	55.93 ± 2.61^c^	53.49 ± 1.72	47.57 ± 1.97	**0.037**
Hydroxyproline	10.77 ± 0.62^c^	9.75 ± 0.53	7.99 ± 0.68	**0.019**
Ornithine	31.84 ± 2.31	33.20 ± 1.75	32.33 ± 1.64	0.874
Citrulline	27.44 ± 1.36	27.49 ± 0.78^a^	23.61 ± 0.97	**0.015**

Values represent mean ± SEM (µmol/L).

^a^NLDI group is significantly different from DT group.

^b^LI group is significantly different from NLDI group.

^c^LI group is significantly different from DT group.

### Plasma glucose, insulin and triglyceride response to dairy ingestion

**Fig. 2:**
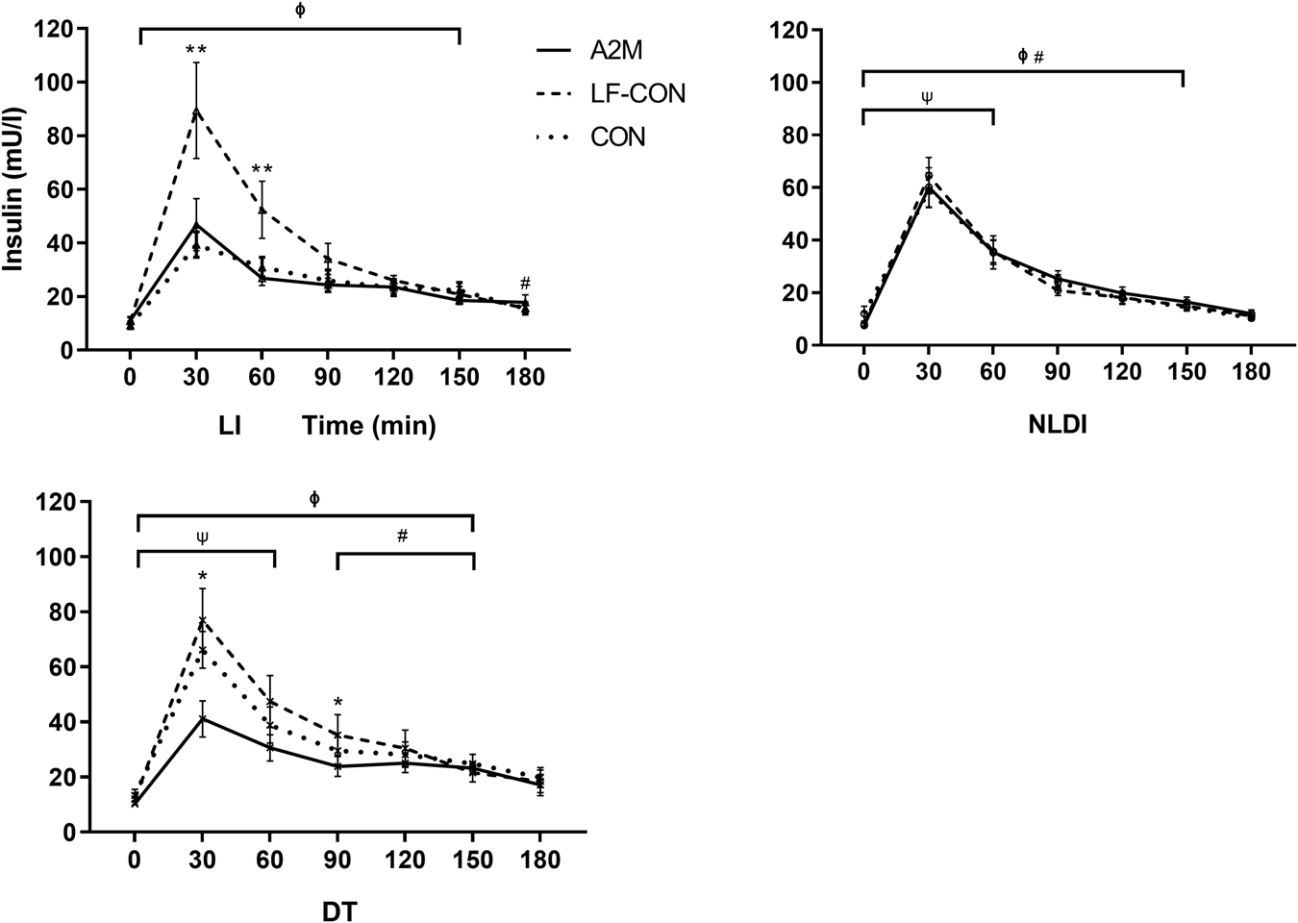
Postprandial plasma insulin levels. The figure shows postprandial plasma insulin levels for each of the three participant groups (NLDI: non-lactose dairy intolerance, LI: lactose intolerance, and DT: dairy tolerant) in response to the different types of milk (A2M: a2 Milk^TM^, LF-CON: lactose-free conventional milk, and CON: conventional milk). Values are presented as mean ± SEM. ** values are significantly different in response to LF-CON than both A2M and CON (*P* > 0.05); * values are significantly different in response to LF-CON than A2M only; φ: postprandial values are significantly higher than baseline in response to LF-CON; #: postprandial values are significantly higher than baseline in response to A2M; ψ: postprandial values are significantly higher than baseline in response to CON.

Postprandial glucose and triglyceride levels were unaltered from baseline values throughout the study period and did not differ between the groups following any of the milk treatments (data not shown). For postprandial insulin we observed an interaction (time × group × milk-type) (*P* = 0.018, Fig. 2). The LI group had higher insulin concentrations at 30 and 60 min after LF-CON, as compared to both A2M and CON (*P* < 0.05).

### Plasma amino acid response to dairy ingestion

**Fig. 3:**
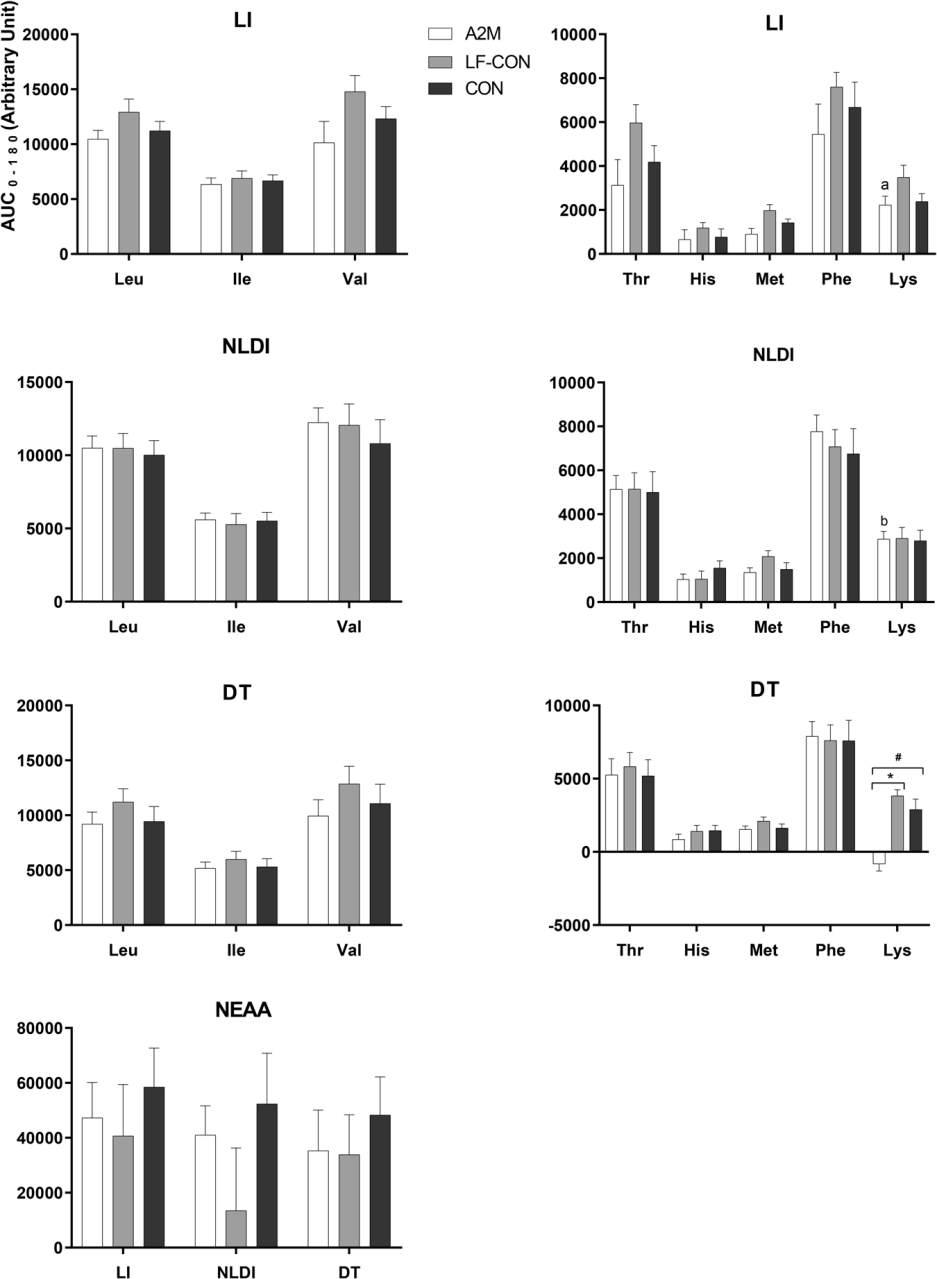
Postprandial plasma amino acid responses. Differences in baseline-adjusted incremental area under the curve (iAUC: 0–180 min) of amino acids between the groups (NLDI: non-lactose dairy intolerance, LI: lactose intolerance, and DT: dairy tolerant) in response to milk varieties (A2M: a2 Milk™, LF-CON: lactose-free conventional milk, and CON: conventional milk). Values are presented as mean ± SEM. a and b indicates the iAUC_0-180_ of AA significantly differ between LI and DT, and NLDI and DT group corresponding to the milk types, respectively (*P* < 0.05). * indicates differences are significant between the two milk types within the group (*P* < 0.05); ^#^ indicates differences are significant between the A2M and CON milk within the group (*P* < 0.05).

No postprandial differences in AA concentrations were observed between the groups in response to the milk treatments with no interaction evident (time × group × milk-type) (*P* > 0.05 for all AA, respectively). As depicted in Fig. 3, in the LI participants, there was no difference in the postprandial iAUC_0-180_ responses for the BCAA, essential amino acids (EAA) and non-essential amino acids (NEAA), between all milk types (*P* > 0.05 for all AA, respectively). Similarly, for the NLDI participants, milk type did not alter the iAUC_0-180_ for the BCAA or EAA. Though non-significant (*P* > 0.05), there was an apparent suppression of the iAUC_0-180_ for the total NEAAs in response to lactose-free milk in the NLDI group. For the DT participants, there was no difference in iAUC_0-180_ for all BCAA and NEAA to the differing milk types (*P* > 0.05 for all AA, respectively); however, amongst the EAA, lysine demonstrated a suppressed iAUC_0–180_ following A2M ingestion, relative to the other two milk types (*P* < 0.05).

When pairwise comparisons were made between the participant groups, there were no differences in the postprandial iAUC_0-180_ for the BCAA, EAA and NEAA between the groups in response to the milk types. Further, of the EAA, the suppressed iAUC_0–180_ of lysine observed in the DT group following A2M ingestion was not reflected in the LI or NLDI group (*P* < 0.05 for both groups).

## Discussion

This study investigated whether the digestion of milk proteins, measured as the postprandial iAUC_0-180_ of plasma AA, would be altered by either lactose intolerance, or a more general dairy intolerance, in response to three different types of bovine milk. Of the two groups with dairy intolerance, lactose was the known trigger in the LI group; however, the trigger for the intolerance in the NLDI group is unknown and was suspected (but not confirmed in the current study) to be due to the presence of A1 β-casein in milk. Protein digestion kinetics in DT individuals was selected to provide a comparable basis of reference. In this study there was no evidence of altered digestion of milk proteins in LI individuals following ingestion of lactose-free milk (LF-CON), compared to either of the lactose-containing milks (CON or A2M). Similarly, for the participants identified as having NLDI, the presence or absence of A1 β-casein in the milk had no impact on postprandial AA responses, but removal of lactose showed a trend toward suppression of the circulatory appearance of NEAAs. Therefore, neither LI nor NLDI appear likely to significantly impact on the protein digestion of differing forms of bovine milk.

In this study, participants were recruited in the basis of identified LI or a self-reported NLDI that included dairy avoidance and self-reported adverse symptoms, but did not include lactose malabsorption. The study also included subjects who reported no adverse symptoms, who regularly consumed dairy containing foods and beverages and who were not lactose intolerant (DT). As we previously reported, the LI and NLDI individuals experienced considerable digestive discomfort compared to the DT group after milk ingestion [16], although this differed by milk type. Thus, GI function was likely altered, but the results of the current study suggest this was not in a manner that compromises protein digestion. In the NLDI group, because the absence of a distinct difference in GI symptoms following ingestion of A2M and CON was reported [16], the same null finding in AA is perhaps unsurprising, and suggests that the A1 β-casein content of milk is not a major determinant of protein digestion differences in this group.

An interesting observation of the current study was the differences in overnight fasted AA concentrations between the participant groups. Relative to the LI participants, concentrations of histidine, arginine, tyrosine, glutamic acid, taurine, hydroxyproline and citrulline were all lower in the DT participants. The NLDI individuals tended to have concentrations closer to those of the DT group. The reasons for these differences are unknown, as are the metabolic consequences. Recent interest is increasing regarding the role of the gut microbiota as a substantial driver of circulating metabolites, including AA [23]. Thus, the observed differences may reflect a relationship between fasting plasma AA levels and altered microbiome composition and function. For example, differences in the microbiome population alter glutamic acid [24], cysteine [25], and tyrosine [26] concentrations in circulation. There is, however, little evidence of altered gut microbiome in LI individuals, although modulating the microbiome of individuals with LI can modify the adverse digestive response following a lactose load [27, 28]. We do not have any data on the baseline microbiome of these individuals, so cannot address this further based on the current study.

The DT group also exhibited a similar digestive response to the three milk types. This is somewhat surprising given that the replacement of only one AA in A2 β-casein (which is the form present in A2M) results in structural differences including formation of smaller casein micelles with enhanced chaperone activity and reduced hydrophobicity during digestion [29]. Furthermore, a more porous microstructure with thinner protein strands resulted in weaker gel structure in fermented A2M compared to CON [15], which would be expected to result in a more easily digestible protein from A2M than CON. In line with these observed differences due to dissimilar physicochemical properties and previous evidence of A1 β-casein induced delayed gastric emptying in experimental models [8] and humans [10], we also expected that A2M relative to CON would support faster digestion and subsequent absorption of proteins.

Furthermore, consistent with previous studies [30, 31], a higher concentration of free AA measured in lactase-treated milk suggests extensive proteolysis of milk protein (particularly β-casein) during storage of LF-CON. Though lactose-free dairy products are becoming more mainstream [32, 33], no data are available on associated structural modifications such as gelling and subsequent impact during digestion; however, because results from experimental models have shown that abundance of free AA is an important determinant of protein digestion kinetics [34], a higher postprandial AA response from ingestion of LF-CON than CON was expected. Individuals in the DT group exhibited a suppressed lysine response following A2M compared to LF-CON and CON. There is prior evidence of blocked lysine during UHT treatment of milk [35], that can restrict subsequent absorption; however, the observations are inconclusive as lysine absorption was not impacted in the LI and NLDI groups.

To the best of our knowledge, this study is the first to report on milk protein digestion in LI and NLDI individuals. Analysis was only made of indirect correlates of protein digestion as reported in other studies [36, 37]; the use of stable isotope analysis to more precisely measure exogenous AA appearance [38] may be required in future studies. Further, no measurement of GI transit was undertaken. Non-invasive techniques including GI transit scintigraphy, the SmartPill™ motility testing system, or non-white light capsule endoscopy could be applied to quantify GI tract transit in the bowel [10], and to detect gut abnormalities through computer-aided diagnostic imaging and sensing technologies [39]. Invasive techniques such as serial biopsies and special histological evaluation may be required to detect inflammation associated with intolerance which is not visible on endoscopy [40].

All milk types used in the study were UHT and were from single batches. UHT is known to alter milk protein structure with greater β-lactoglobulin denaturation and complex formation with the casein component [41]. This may impact on the digestibility of the milk and previously it has been shown that UHT milk is more rapidly digested than non-heated microfiltered milk [42]. Thus, while in the current study we have shown that LI and NLDI do not markedly alter milk protein digestion, the rate and extent of protein digestion may vary with the use of differing milk processing technologies, including processing temperature and storage condition-induced modifications [43, 44].

Background characteristics of participants, such as age and sex, can influence circulatory concentrations of AAs [45]. Ageing results in many physiological and psychological changes including oral malfunction [46], altered taste and smell [47], decreased appetite [48] and hormonal imbalance [49]. In addition to a reduced dietary intake, altered digestive capacity in older adults may be responsible for differences in circulatory AAs. Previous research from our group has shown that digestion and absorption of proteins from a mixed meal was delayed in older adults as compared to younger adults [50]. Further, sex-specific patterns of AAs have been observed in relation to the use of AAs as body fuel [51], which may cause differences in circulatory concentration of AAs. To avoid the confounding effects of these differences on circulatory concentration of AAs, we included only young female subjects in the current study. The limitation of this approach is that it restricts the generalisability of the outcome. Further studies are therefore required to confirm these observations in other populations such as young male (aged 20–30 years) and older male and female participants (aged 60–75 years).

In addition to measuring the concentrations of 22 AAs, several additional pre-specified secondary end-points (previously described [16]) were measured, which are not reported here. While the data reported here were adjusted for multiple comparators as stated for the post hoc tests in the methods (i.e., time, groups, and milk-types), the multiple analyses were not adjusted for unreported secondary end-points, which may limit the study’s power for such detailed analysis.

This study did not identify the trigger for milk-induced discomfort in the NLDI individuals (previously described [16]). It is possible that the symptoms observed in this group are due to a sensitivity to any visceral stimulation, and not to the milk per se. This could be a genuine effect of the relatively large volume of milk consumed, or even a nocebo effect where the symptom is expected by the participant. There is also the possibility that more complex GI disturbances and diseases, including Irritable Bowel Syndrome and Coeliac disease, are present within the LI and NLDI participants [52, 53]. No additional screening or clinical examination was made to confirm the absence of these conditions, however all participants were healthy and not receiving specialist gastrointestinal-related medical care. Unphysiologically large doses of lactose and milk were provided which do not represent usual intake. Although smaller doses are recommended for diagnosis of clinically relevant lactose intolerance [54], high doses are reasonable in the context of this study because less milk would have likely reduced the sensitivity of AA measurements. Based on previous evidence [55], it can be anticipated that at smaller doses (e.g., 12 g of lactose, or one 250 ml glass of milk), the extent of GI disturbances would be further minimised. It is therefore unlikely that differences in protein digestion would be present in LI individuals with the ingestion of smaller milk volumes.

In summary, milk protein digestion, measured on the basis of changes in circulating AA, was not altered in young females who have either LI or who avoid regular dairy consumption on the basis of self-reported dairy intolerance that is not due to lactose malabsorption (NLDI). Other than a suppressed lysine level in the DT group in response to A2M (compared to the other two milk types, and the other subject groups), there were no differences in the iAUC_(0-180)_ for all measured AA between participant groups (defined by their tolerance to dairy), or in response to the three types of commercial bovine milk tested.

## Data Availability

Additional data are available from the corresponding author on reasonable request.
